# Reducing risks of antibiotics to crop production requires land system intensification within thresholds

**DOI:** 10.1038/s41467-023-41258-x

**Published:** 2023-09-29

**Authors:** Fangkai Zhao, Lei Yang, Haw Yen, Qingyu Feng, Min Li, Liding Chen

**Affiliations:** 1 School of Ecology and Environmental Sciences, Yunnan University, Kunming, 650500 China; 2 State Key Laboratory of Urban and Regional Ecology, Research Center for Eco-Environmental Sciences, Chinese Academy of Sciences, Beijing, 100085 China; 3 University of Chinese Academy of Sciences, Beijing, 100049 China; 4 School of Forestry and Wildlife Sciences, Auburn University, Auburn, AL 36849 USA; 5 Environmental Exposure Modeling, Bayer U.S. Crop Science Division, Chesterfield, MO 63017 USA

**Keywords:** Environmental impact, Sustainability, Agriculture

## Abstract

Land system intensification has substantially enhanced crop production; however, it has also created soil antibiotic pollution, undermining crop production. Here, we projected soil antibiotic pollution risks to crop production at multiple geographical scales in China and linked them to land system intensification (including arable land expansion and input increase). Our projections suggest that crop production will substantially decrease when the soil antibiotic pollution risk quotient exceeds 8.30–9.98. Land systems explain most of the variability in antibiotic pollution risks (21–66%) across spatial scales. The convex nonlinearities in tradeoffs between antibiotic pollution risk and crop production indicate that vegetable and wheat production have higher thresholds of land system intensification at which the risk–yield tradeoffs will peak than do maize and rice production. Our study suggests that land system intensification below the minimum thresholds at multiple scales is required for acceptable antibiotic pollution risks related to crop yield reduction.

## Abstract (web summary)

Crop intensification has increased agricultural production albeit with an increase in field antibiotic pollution. Here, Chen et al. project how antibiotic pollution undermines production and how intensification needs to be kept below a threshold.

## Introduction

Since the onset of the Anthropocene, human activities have driven substantial socioeconomic development; however, they have also created significant environmental risks from food security to chemical pollution, which must be managed^1–3^. As an emerging issue, antibiotics are used worldwide to control microorganisms (e.g., for human disease prevention and animal growth promotion)^4,5^. Unfortunately, antibiotics are generally emitted into the soils during their manufacture, use, and disposal, and agricultural practices have been one of most important sources of antibiotic emissions, such as manure fertilization and wastewater irrigation, which contain a large amount of antibiotics^2,6^. Soil antibiotic pollution thereby increasingly undermines the health of agricultural ecosystems, such as by inhibiting plant growth, disturbing soil functioning, and ultimately reducing crop production^7,8^. Given the increase in human emissions of antibiotics, risks to crop production caused by antibiotic pollution are expected to increase in the future^4,5^. Thus, managing antibiotic pollution risks is crucial for maintaining crop production and sustainability^9^.

Although the antibiotic footprint has received considerable attention^10^, the repercussions of antibiotic dispersion in the soil remain largely unknown. In both developed and underdeveloped countries, it is difficult to determine soil antibiotic pollution risks on a massive scale, which exacerbates the lack of a robust analysis that allows us to synthesize the risks to crop production. This has proven to be a serious scientific challenge that limits our understanding of the linkages between antibiotic pollution and food security. Several studies have attempted to simulate the spatial characteristics of antibiotics in soil^11,12^. However, these approaches are limited in that they have restricted geographic extents and do not directly characterize the risks to crop production^13,14^.

It has been highlighted that land use plays a crucial role in soil antibiotic pollution^15,16^; however, the causes of antibiotic pollution risks are still substantially challenging, partially because land systems change over a vast range of spatial scales. Land system intensification, particularly arable land expansion and increased agricultural inputs (e.g., increased fertilization and irrigation), which seeks to increase crop yields and associated economic returns, can substantially exacerbate soil antibiotic pollution in parallel with improvements in crop yields^17,18^. For instance, high manure application rates increase the loading of antibiotics into soil and result in serious soil antibiotic pollution in a given region^19^. It has also been recognized that larger areas of arable land have higher likelihoods of receiving agriculture-originated antibiotics^15^. Thus, understanding the responses of antibiotic pollution to land system regimes and the thresholds for sustainable land system intensification is greatly beneficial for corresponding risk management. Recent studies suggest that land systems distinctly affect antibiotic pollution risks from regional to global scales^15,20^. Controlling human societies to reduce antibiotic pollution at multiple scales requires sustainable land system intensification, including modifications to land use patterns and land management practices^21^. How land system intensification considering different spatial scales affects antibiotic pollution is the key question in addressing the risks to crop production; however, few efforts have attempted to bridge these gaps.

Filling these gaps can further promote sustainable socioecological outcomes of land system intensification, balancing crop production and soil antibiotic pollution. This study aims to address three questions: (i) Do antibiotic pollution risks reduce crop production at a broad scale? (ii) How do land systems affect soil antibiotic pollution risks with changing spatial scales? (iii) How can land system intensification be managed to balance antibiotic pollution risk and crop yield based on the potential thresholds where risk–yield tradeoffs take their maximum values? To address these questions, we examine the scale-dependent effects of land system intensification on soil antibiotic pollution risk by taking China as a case study, which is one of the greatest consumers of antibiotics worldwide and is exposed to antibiotic pollution^20^. The risk quotient (RQ, unitless) of antibiotics to arable crop growth is estimated to characterize soil antibiotic pollution risk, and the risk quotient is defined as the extent to which environmental concentrations surpass their no-effect concentrations^22^. We first predict the risk associated with antibiotics on a broad scale using machine learning logarithms and analyze the relationships with maize, rice, wheat, and vegetable production. According to the development Kuznets curve hypothesis, which describes nonlinear relationships between sustainable development and inequality (e.g., economy and environmental elements)^23^, we examine inverted U-shaped relationships between land system intensification and antibiotic pollution risk to crop production when spatial scales change (that is, decrease or increase in the size and extent of space, Supplementary Note 1, Supplementary Figs. 1 and 2). We further explore the thresholds of land system intensification at which antibiotic pollution risk substantially increases. On the basis of these analyses, we provide guidance for the development of tailored strategies to balance antibiotic pollution risk and crop production.

## Results

### Predicting the risks of antibiotics to crop production

**Fig. 1:**
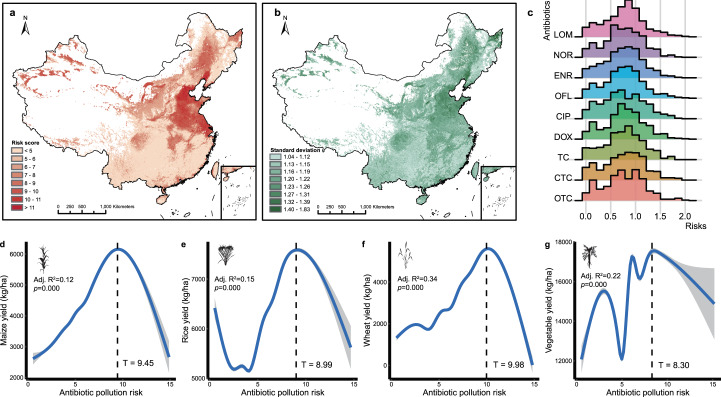
Risks of antibiotics in soil to crop production in China. **a**, **b** Maps of the cumulative risks associated with target antibiotics and uncertainties associated with the predictions. Cumulative risk quotients for nine target antibiotics were calculated per grid on the basis of the concentration addition principle^24^. The maps of China used in this study were adapted from the data released by the National Administration of Surveying, Mapping and Geoinformation of China (http://www.sbsm.gov.cn; review drawing number: GS(2020)4619). **c** Density distribution of risks associated with each antibiotic; the ridge line plots the estimated density from predicted data. **d**–**g** Convex nonlinearities in the relationships between crop yields and antibiotic pollution risks. The lines (regression lines) and shaded areas (95% confidence intervals) were estimated by generalized additive models, and adjusted (Adj.) R^2^ and two-sided *p* values of the t-statistic for each model are provided. The *T* values (thresholds of antibiotic pollution risk for crop yield reduction) were estimated by the “*segmented*” package in R according to the significant changes in regression slopes^69^. All silhouettes are from PhyloPic (https://www.phylopic.org/).

To quantify the risks to crop production in each geographic grid cell, we modeled the RQs of each target antibiotic in soil using ensemble random forest (RF) models fed with georeferenced datasets (Supplementary Fig. 3). The differences between the modeled and validated RQs were generally less than 1, with correlation coefficients ranging from 0.48 to 0.68, demonstrating the good prediction performance of our models (Supplementary Fig. 4). This procedure allowed us to clarify soil susceptibility to antibiotic pollution. To elucidate the extent of pollution caused by antibiotic mixtures, we calculated the cumulative RQs for the ecosystem in each grid cell on the basis of the concentration addition principle^24^. The average cumulative RQ of antibiotics in the Chinese soil environment was 6.1 ± 2.1 (Fig. 1a). The cumulative RQ was particularly high in central and eastern China, which experience considerable exposure to antibiotics. The relative uncertainty (ratio of standard deviation to mean) across the ensemble model was ±26% of the predicted data on average, but areas of substantial uncertainty existed. Huang–Huai–Hai and the Northeast Plains, which are the main grain production areas accounting for more than one fifth of the agricultural output of China, exhibited the highest uncertainties (Fig. 1b). This suggests that anthropogenic activities possibly caused a wide range of antibiotic pollution risks in soil. An RQ > 1 indicates that target antibiotics pose serious risks to crop yield, which is recognized by EU risk assessments^25^. Our model estimated that ~11.4% of the land was contaminated by more than one antibiotic compound. Among the target antibiotics, ofloxacin accounted for the most pronounced risk, with RQs of 0.76 ± 0.37 (Fig. 1c).

We also asked whether soil antibiotic pollution could influence crop production on a broad scale. Our analysis showed that the soil antibiotic risk consistently imposed nonlinear effects on maize, rice, wheat and vegetable production (Fig. 1d–g). Generally, when the cumulative RQ of soil antibiotic pollution exceeded 8.30–9.98, the yields of these crops substantially decreased and were even lower than the crop yields when the risks were zero. This indicates that serious antibiotic pollution could partially offset the positive effects of human activities on crop yield increase.

### Scale-dependent land system effects on the risks of antibiotics in soil

**Fig. 2:**
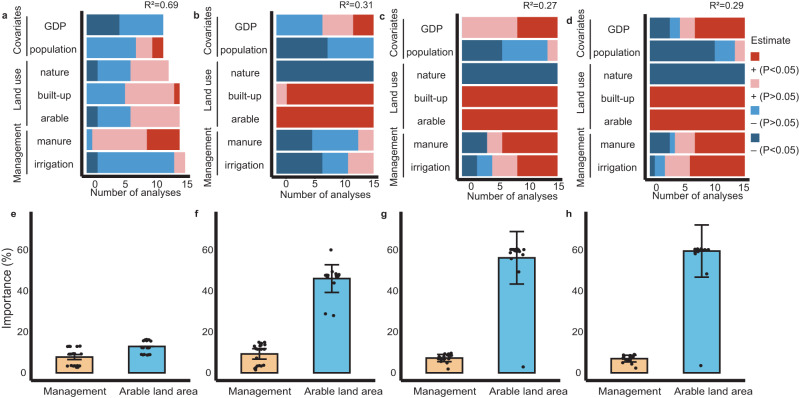
Scale-dependent effects of land system on the risks of antibiotics in soil. **a**–**d** Number of analyses (20 full-parameter analyses with different datasets: segments from the upper and lower limits of the same data were excluded) with significantly positive (+, red) or negative (−, blue) or nonsignificantly positive (+, light red) or negative (−, light blue) relationships with antibiotic pollution risks based on linear regressions. The two-sided *p* values estimated by the t-statistic are used to identify the statistical significance when their values are less than 0.05. Land use was measured by the area proportions of arable, built-up, and natural lands. Land management was measured by manure application rate and irrigated area. The average R^2^ values for 20 full-parameter analyses are presented. **e**–**h** Contributions of arable land use and management to soil antibiotic pollution risks; the columns and error bars indicate mean contribution and standard deviation (*n* = 20 subdatasets). The points on the columns show the data distribution. The plots from left to right are the results of watershed level 1, level 2, level 3, and level 4. Level 1 corresponds to large watersheds.

Since the local-specific relationships between land systems and antibiotic pollution risks might be hidden to a large extent, we evaluated scale effects by reducing the scale extent (that is, the proportion of areas selected along the human-impact gradient, Methods and Supplementary Note 1). We excluded the segments from the lowest or highest ends of the human-impact gradient to explore how the relationships between land systems and antibiotic pollution risks were altered in watersheds subjected to high or low human footprint pressures. At the large watershed level, the change in scale extent had divergent effects, suggesting that the spatial extent strongly modulated human impacts on antibiotic pollution risks (Supplementary Figs. 5 and 6). Nevertheless, spatial extent changes had limited influences on the land system–antibiotic pollution relationships at small watershed levels. By combining the 20 analyses with different spatial extents, our results suggested that antibiotic pollution risks strongly correlated with land systems (including both land use and management) in most cases (100%, particularly at the small watershed level), while they were less correlated with population and economic indicators (Fig. 2a–d), indicating a more fundamental role of land systems in regulating antibiotic pollution risks across scales. At the small watershed level, management (particularly manure application) increased the risk levels in 16 of 20 analyses (Fig. 2d). Shifts in land systems, such as land use composition and management practices, enable us to capture the likelihood of soil antibiotic pollution risks across scales. Given the complementarity between land use and management, they showed opposite scale-dependent contributions to the RQs of antibiotics (Fig. 2e–h). With downscaling, the contribution of arable land to antibiotic pollution increased from 12.9 ± 3.2% to 59.5 ± 12.7%. These results indicate that the land system–antibiotic pollution relationships were scale-dependent, suggesting the necessity of different land system management strategies to control antibiotic pollution risks.

### Risk–yield tradeoffs through land system intensification

**Fig. 3:**
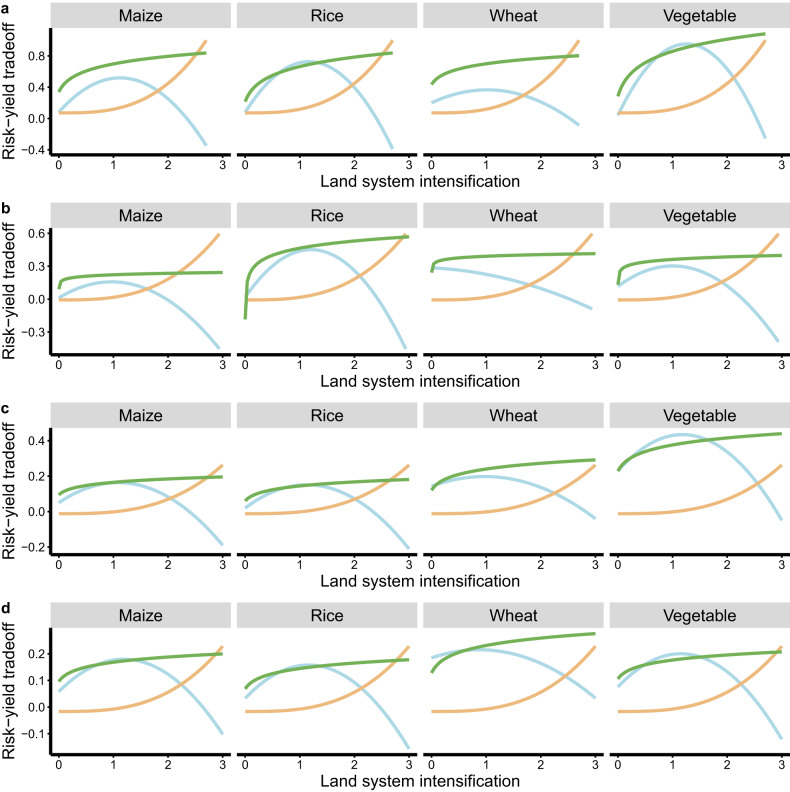
Tradeoffs between antibiotic pollution risk and crop yield with increasing land system intensification. Nonlinear relationships between risk–yield tradeoffs and land system intensification. The green and yellow lines are constraint lines of scaled crop yields and scaled antibiotic pollution risks (RQs) based on the scattered point clouds (see Methods). An example of the constraint lines and scattered point clouds is presented in Supplementary Fig. S8. The tradeoff (cyan lines) is defined as scaled yield minus scaled RQ, which represents that benefits for crop yield are higher than risks when the value is higher than zero (Supplementary Fig. 9). Land system intensification is estimated by summing the standardized scores of land use composition (i.e., proportion of arable land) and management (including manure application rate and irrigation area proportion) within a specific watershed. **a** Level 1 watershed. **b** Level 2 watershed. **c** Level 3 watershed. **d** Level 4 watershed.

Changes in the risk–yield relationship with land system intensification showed that a tradeoff existed between antibiotic pollution risks and crop yields (Fig. 3). The area between the crop yields and risk curves indicates the potential to improve land system intensification. According to the risk–yield tradeoffs, which are defined as the difference between scaled yield and risk, vegetable yield had a higher improvement potential than maize, rice, and wheat, and it had higher thresholds of land system intensification across scales. The results demonstrate that although land system intensification can improve vegetable production, its benefit–risk ratio decreased when land system intensification crossed the thresholds; that is, the benefit increase might be lower than the risk increase.

**Fig. 4:**
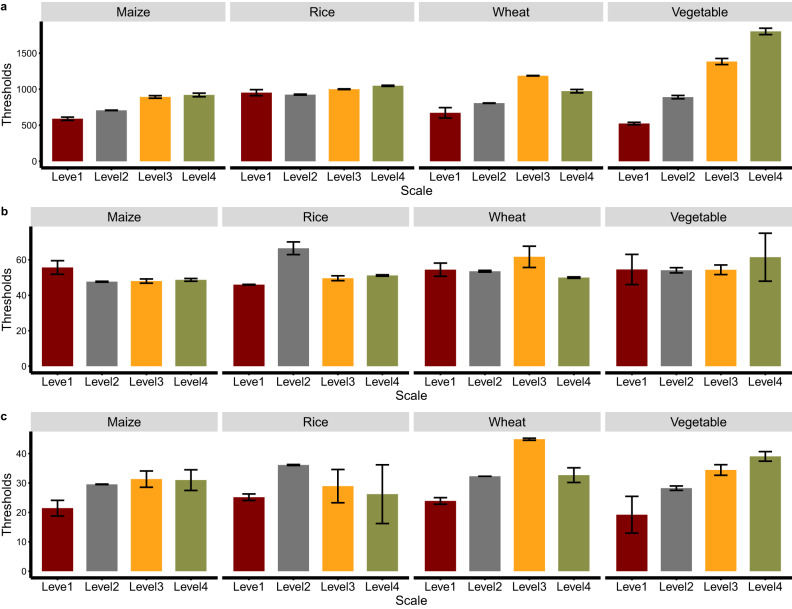
Thresholds of land system intensification for risk–yield tradeoffs across scales. **a** Thresholds for manure fertilization (kg N/km^2^/yr). **b** Thresholds for irrigated area proportion (%). **c** Thresholds for arable land proportion (%). The development Kuznets curves are used to describe the relationships between land system intensification and risk–yield tradeoffs, and inverted U-shaped relationships generally exist (Supplementary Fig. 7). A threshold of land system intensification is identified when the inverted U-shaped curve has a slope value of zero. The columns and error bars indicate mean values and standard errors, which were estimated by uncertainties of regression parameters (*n* = 17 for level 1, *n* = 177 for level 2, *n* = 414 for level 3, *n* = 818 for level 4).

To manage land systems, their effects on risk–yield tradeoffs were evaluated on different scales via the moving-window approach. The convex nonlinearities in the effects of land management on risk–yield tradeoffs were widely observed for four arable crops across scales (Supplementary Fig. 7). These convex nonlinearities suggested that there were thresholds of land system intensification where risk–yield tradeoffs peaked (Fig. 4). Our analysis showed that vegetable and wheat production had higher thresholds (vegetables: 523.5–1801.6 kg N/km^2^/yr, wheat: 672.1–1186.9 kg N/km^2^/yr) of manure fertilization than maize and rice production, particularly at small scales. For irrigated areas, vegetable and wheat production generally also had high thresholds (vegetables: 54.4–61.5%, wheat: 50.0–61.7%) at small scales, while rice production had high thresholds at large scales (46.0–66.5%). Given the importance of arable land to antibiotic pollution risk (Fig. 2e–h), we also analyzed the arable land area thresholds for risk–yield tradeoffs. Vegetable and wheat production also had higher thresholds (vegetables: 34.4–39.1%, wheat: 32.7–44.9%) than maize and rice production at small scales. These results suggest that vegetable and wheat production seem to have high resistance to antibiotic pollution at small scales.

## Discussion

During the Anthropocene era, human demands and land use intensity have increased. Throughout history, humans have modified the soil ecosystem to obtain life-sustaining resources. This has recently resulted in negative environmental effects, such as antibiotic pollution, which must be managed to protect crop production^26^. This study found that using an ensemble random forest model fills the gaps in information on antibiotic pollution in China and expands previous regional-scale risk assessments, which were limited by data availability and spatial scale^12,27^. We first investigated the susceptibility of crop production to soil antibiotic pollution on a broad scale (Fig. 1). The results revealed that antibiotic pollution posed a widespread risk to the crop production of China, which has similar geographic patterns to land system intensification (which showed increasing trends from west to east), as partially substantiated by a national-scale survey^28^. The prediction revealed that ofloxacin posed a higher risk to the soil ecosystem than other target antibiotics (Fig. 1c), which is similar to its risk priority calculated by ranking its prevalence, persistence, bioaccumulation, and toxicity^29^.

Here, we quantitatively investigated human impacts on risks to soil antibiotic pollution across a broad range of scales (Fig. 2). Despite the importance of soil and climatic conditions, human activities are the dominant predictor of antibiotic pollution risks^30^. Among various anthropogenic factors, land systems, which are terrestrial socioecological systems that interact with human and environmental subsystems^31^, have been demonstrated to affect antibiotic pollution risks on multiple spatial scales^14,30^. Since the intensification of the land system, land use composition and management intensity have been the predominant factors affecting antibiotic pollution risks across spatial scales instead of population and economic systems. Weak correlations between population density and antibiotic pollution in soil were also demonstrated in previous studies^32,33^. These findings suggested that population and economic systems likely did not directly affect soil antibiotic pollution but rather had indirect effects on soil antibiotic resistance globally^20^. This is because antibiotics were not directly discharged into soils by population and economic systems but rather were generally released through land systems; that is, human impacts on soil antibiotic pollution risks were ultimately mediated via land systems. For instance, urban sewage sludge (or wastewater) containing antibiotics is usually not applied on urban land and is transported to agricultural land as fertilizer or other neighboring sites^34^. Given that wastewater is one of the main sources of antibiotics in agricultural soils, the fact that the irrigated area proportion was positively correlated with antibiotic pollution risks supported our hypothesis, that is, increased inputs (i.e., land system intensification) enriched antibiotics in soils. Moreover, population and economic growth would significantly promote land system intensification and thus increasingly introduce antibiotics into soils. As an example, it has been demonstrated that the expansion of arable lands, which are treated with antibiotic-containing fertilizer, could result in antibiotic pollution to more land^30,35^.

Interestingly, we found that the effects of land systems on antibiotic pollution risks were influenced by spatial scale in this study, indicating that the cumulative effects of land systems declined with increasing scales (Fig. 2). Land management, rather than land use composition, primarily explained antibiotic pollution risks on a large spatial scale. This ‘scale dependence’ possibly arises for two major reasons. First, the spatial scale at which data are analyzed will substantially change the data variability. Despite the expansion of human-dominated landscapes, natural land (including forest and grassland) still accounts for most of the Earth’s land surface (for example, built-up lands account for less than 0.7% of the total land area on Earth)^36^. From the viewpoint of a broad scale, the variability in land use is generally lower than the variability in management among watersheds, and it is the latter that causes the differences in antibiotic pollution risks. Second, human use that triggers antibiotic discharges can vary dramatically across spaces and respond differently to human activities across scales. Bu et al.^37^ found an inconsistent pattern of antibiotic use with scale change, suggesting that some western regions of China possessed low antibiotic use on a large scale but high use on a small scale. Therefore, scale change will modify the spatial patterns of management-induced antibiotic discharges (e.g., reuse of wastewater and manure excreted by humans).

One of the facts about land systems with regard to sustainability is that land systems often create tradeoffs between social equality (i.e., crop yield) and environmental safety (i.e., antibiotic pollution risks) rather than win‒win outcomes^17,31^. Based on a simple assumption, land system intensification may linearly increase crop yield. However, we observed nonlinear trends in crop yield increase with increasing land system intensification, suggesting that benefits for crop yields were partially offset by soil antibiotic pollution. This can be confirmed by our finding that antibiotic pollution reduced crop yields when its cumulative RQ exceeded 8.30–9.98. Given the substantial effects on risk–yield tradeoffs, sustainable land system intensification may offer an alternative for risk management. It can be expected that watersheds consisting of a higher proportion of arable land experience more severe antibiotic pollution^7^. Thus, reducing the area proportion of arable land can potentially mitigate antibiotic pollution. However, it is unreasonable to crudely decease areas of human-dominated landscapes in a watershed to reduce soil antibiotic pollution risks, which might also reduce socioeconomic outcomes (e.g., crop yields). Intensification of the land system is now one of the main agendas benefiting socioeconomic development across and within human societies and thus is typically associated with benefit–risk tradeoffs^17^. In our study, the tradeoffs between crop yield and antibiotic pollution risk show that the high priority afforded to sustainable land system intensification efforts is necessary to achieve the expected likelihood of win–win outcomes, that is, crop production and risk control.

There were many difficulties in decision-making for sustainable land system intensification. We set lenient limits for land system intensification to control soil antibiotic pollution risks, suggesting that it should not exceed these thresholds to balance the risk–yield tradeoffs. Based on the development Kuznets curve hypothesis^23^, we elucidated the inverted U-shaped relationships between land system intensification and risk–yield tradeoffs. Thus, the thresholds where the risk–yield tradeoffs take their maximum values fall on the inverted U-shaped curves. These thresholds are comforting for decision-makers as they can capture the desired information for land system management. When land system intensification exceeded the thresholds, we will risk a reduction in crop production; that is, the relative benefits for crop yields decreased and antibiotic pollution risks increased (Fig. 3). Waiting to cross these thresholds could avoid a situation in which antibiotic pollution risks exceed benefits for crop yields.

It is often not clear whether these win–win outcomes are achieved across different scales because land system–antibiotic pollution relationships are scale-dependent. Further complexities arise because the scales at which policy decisions are made often do not match the scale at which human activities matter^31^. We attempted to account for the scale effects, as scale changes can completely shift statistical supports. Our analysis revealed that vegetable and wheat production had a higher likelihood of achieving win–win outcomes at small scales than maize and rice production (Fig. 4); thus, policymakers, farmers, and other actors should pay more attention to small-scale crop structure changes. When crop production is not greatly compromised, land system intensification within small-scale watersheds should be carefully considered according to the thresholds for risk–yield tradeoffs to protect crop production with low environmental costs. Overall, the findings of this study and others^38^ suggest that land system intensification and its win–win outcomes are urgent issues limiting sustainable development, and more efforts considering safe limits should be undertaken.

Our study goes beyond previous work on the risk assessment of antibiotic pollution in soil on multiple spatial scales. However, the following uncertainties and limitations need to be further considered. (i) The antibiotic pollution risk presented in this study may be overestimated, mainly because the risk assessment model assumed maximum exposure and minimum tolerance of nontarget organisms to represent the worst scenario; nonetheless, the estimated risk can be a good reference for regulatory agencies in making critical decisions because the worst scenario represented soil antibiotic pollution risks that can maximally lead to negative changes (i.e., crop yield reduction) in land systems. (ii) This study only considered the exposure risks to crops, but the spread of antibiotic-induced resistance was not included. In addition, biotic factors seem overlooked in the present work, although soil microbes or plant microbiomes are believed to play crucial roles in the relationships between antibiotic pollution and crop yield. Unfortunately, long time-series datasets of biotic factors (e.g., soil microbes and plant microbes) are lacking at a broad scale. It has been shown that the diversity of soil microbes and plant microbes is significantly correlated with soil organic carbon, precipitation, and temperature^39,40^, which were considered in this study. The relationships of these factors with microbes might partially reduce the uncertainties caused by the absence of microbes in our models. (iii) We assumed the nonexistence of target antibiotics that were not reported; however, these antibiotics might be present but below the detection limit of the adopted methodology. (iv) This study was performed based on modeling results without the inclusion of a validation experiment, and thus lacks a mechanistic understanding. When the universal screening of antibiotic pollution is difficult and not cost-effective, a modeling approach can provide reliable evidence. Several studies have modeled antibiotic concentrations in soil using regression techniques, although with limited modeling accuracy^11,41^. Additional biophysical models and available data should be developed to accurately predict the risks associated with antibiotics in multiple environmental compartments and investigate the mechanisms underlying the socioecological system.

Nevertheless, our findings might enable generalization to other regions or countries. Intensive land systems often result in high soil antibiotic pollution risks, which hinder crop production. Inverted U-shaped relationships between land system intensification and risk–yield tradeoffs were generally observed, and the thresholds of land system intensification, where tradeoffs were maximized, provide a comforting guide for decision-makers aiming to manage land systems based on a balance between crop yields and antibiotic pollution risks. Sustainable land systems are required for antibiotic pollution control to protect crop production. In addition, to achieve sustainability goals, cooperation among innovative and effective policies, implementation strategies, and stakeholder actions for land system sustainability is required for sustainable soil use and sustainable living with low exposure to antibiotic pollution.

## Methods

We collected data on the measured environmental concentrations (MECs) of antibiotics in Chinese soil. Many antibiotics widely occurred in soil, and nine frequently detected antibiotics with wide distribution and high toxicity were selected: tetracycline, chlortetracycline, oxytetracycline, doxycycline, ofloxacin, norfloxacin, ciprofloxacin, enrofloxacin, and lomefloxacin. Then, we evaluated the risks of these target antibiotics to crop production. We also collected data on anthropogenic factors, climatic factors, and soil and vegetation characteristics that could explain the variability in antibiotic pollution risks as predictors. Next, we scaled up the risks associated with target antibiotics in soil using a machine learning model (RF algorithm)^42^. We combined a linear model, generalized additive model, and moving-window approach to describe the relationships between human activities and antibiotic pollution risks on four scales. All modeling and analyses were conducted using R software 4.2.1.

### Data collection

To determine the risks of soil antibiotic pollution, a comprehensive MEC dataset was established. We mainly compiled the publicly available dataset of Zhang et al.^43^, which records antibiotic occurrence and distribution in Chinese soil. This dataset contains georeferenced occurrences for our target antibiotics, considering land use type (urban green space, cropland, grassland, and forestland) and experiment location (coordinates) at a specific sampling time. Concentrations that were ‘not detected’ or ‘below detection limit’ were regarded as zero values. The MECs were converted into standardized units (μg/kg or ng/g). We revisited the data sources of the dataset of Zhang et al.^43^ and found that the measurements of antibiotics were performed with a depth of 20 cm in most cases. When scaling up the risks of soil to antibiotic pollution, we considered a fixed depth value of 0–20 cm.

To expand the existing data, we also collected soil samples from Yunnan and Zhejiang provinces and measured the concentrations of target antibiotics. The soil samples were transported to the laboratory, freeze-dried, and stored at −20 °C until further analysis. The extraction and analysis of antibiotics are detailed in the Supplementary Methods (Supplementary Note 2). The measured antibiotic data were combined with the dataset of Zhang et al.^43^, which comprehensively depicts the MECs of soil antibiotics in most regions of China.

The following selection criterion procedures were performed for our combined dataset to minimize possible bias: (i) we only considered the field samples that were not experimentally treated, such via addition of antibiotics; (ii) soil samples collected from seriously polluted sites (such as hospitals and livestock farms), where high levels of introduced antibiotics might add a layer of uncertainty, were not considered in this study; and (iii) we calculated the Moran’s *I* index to evaluate the bias caused by spatial autocorrelation in our dataset (Moran’s *I* = 0.03, Z score = 0.22, *p* > 0.05), and the results suggested that our dataset does not appear to be significantly different than random distribution. Finally, our selection criteria generated a dataset of 484 locations which is higher than the minimal sample size requirement^20^. Our measured data are available in Supplementary Data 1, and other raw data can be downloaded from *figshare*^44^.

### Risk assessment model

Given the disparities in the tolerance of crop growth to different antibiotics, the risk of antibiotics to crop growth can be better representative of the consequences of antibiotic pollution than their concentration^45^. In this study, risk assessment of antibiotics is performed following the risk quotient (RQ) approach, with RQs for individual mixture components determined from MECs and predicted no-effect concentrations (PNECs). To assess the risks of the target antibiotics, the PNECs were first estimated via the assessment factor approach^22^. PNECs were determined as the ratio of the median effective concentration (EC50) or median lethal concentration (LC50) to the assessment factor (AF = 1000) or the ratio of the chronic no-observed-effect concentration (NOEC) to the assessment factor (AF = 100); that is, PNEC = EC50 (or LC50)/1000 or PNEC = NOEC/100. Owing to the scarcity of available data, the toxicity data of the relevant terrestrial organisms (including soil animals, microbes, and plants) were collected from the literature (Supplementary Table 1), and the lowest PNECs were used for further calculation to represent the worst scenarios. Then, the species-specific RQs of the antibiotics were calculated as the ratio of MEC to PNEC (that is, RQ = MEC/PNEC). For some antibiotics, the PNECs of soil (PNEC_soil_) were estimated from their values in water (PNEC_water_), which were corrected by the soil–water partition coefficient (*K*_d_) via the equilibrium partitioning method (that is, PNEC_soil_ = PNEC_water_ × *K*_d_). To consider the worst scenarios, we defined the risk levels of antibiotic pollution as the highest RQ values. We determined the cumulative risks in soil as the sum of the highest RQs of each target antibiotic.

### Identification of candidate predictor variables

The predictor variables were determined from spatially explicit datasets (Supplementary Table 2). Climatic information on the annual average temperature and precipitation at the specific sampling time was sourced from the CRUTS V4 database^46^. Land use data were released from the Resource and Environmental Science and Data Center (https://www.resdc.cn). Five anthropogenic factors were collected, namely, livestock density (cattle, pigs, and chickens), human population density, GDP, chemical fertilization (nitrogen and phosphorus), and pesticide use, which are linked to socioeconomic development and agricultural activities. Soil properties, which substantially influence the fates and behaviors of antibiotics in soil, were collected as important predictor variables; they included clay content, organic carbon content, bulk density, soil thickness (distance to bedrock), and saturated hydraulic conductivity. We selected the groundwater table depth and normalized difference vegetation index as indicators of leaching and plant uptake capacity. Moreover, we obtained terrain information from the GTOPO30 database^47^, as terrain can influence antibiotic movement on land. To ensure compatibility across datasets and variables, we processed all data at a spatial resolution of 1 km, which was consistent with the land use data. Gridded datasets with higher pixel resolutions were resampled by the mean aggregation method, and datasets with lower resolutions were resampled by simple upsampling (i.e., without interpolation)^48^. The data were extracted according to the experimental site location and sampling year. We assumed that several predictor variables (e.g., soil properties and terrain) had limited temporal changes due to the lack of time-series datasets, while the other predictor variables generally had long time-series datasets from 2000 to 2020. Anthropogenic and climatic factors as well as soil and vegetation characteristics determine the amount of antibiotic residue in soil and the soil susceptibility to antibiotic pollution.

### Scaling-up approach and output maps

An RF model including all of the predictor variables^42^ was fitted to the log-transformed (log_10_) RQs of each antibiotic using the R packages *randomForest*^49^ and *Caret*^50^. All predictor variables were standardized using the Z score. We constrained the RF models by setting the maximum number of allowed trees to 1000. Since the RQ data were modeled using 2020 datasets, cross-validation was performed. The dataset was randomly divided into training and validation sets, corresponding to 70% and 30% of the data, respectively. Finally, the data-trained RF model was applied to the gridded data of predictors to estimate the risk levels of nine target antibiotics on a larger scale. Ecosystems not covered by soil (including bare land and water) were excluded from the analysis based on the land-cover map. An ensemble model for our final predictions and uncertainty analysis was developed via the Monte Carlo approach. Our final model was an ensemble of 500 RF models (that is, the model was separately trained 500 times; each time, a different set of data was selected from our dataset) to generate a distribution of model error on random data^51^. The distribution density of differences between measured and predicted data was determined for each target antibiotic, and the root mean square errors (RMSE) and Pearson’s correlation coefficients between measured and predicted data were used to assess the accuracy of the RF models. We derived the models’ mean prediction across the ensemble models and determined the uncertainties associated with the predictions from the standard deviation. Maps of the distribution of risks related to soil antibiotic pollution, expressed as average RQs, and the corresponding uncertainty maps were derived using the data-trained RF models at a resolution of 1 km.

### Land system indicators

The land system can be manipulated by technologies and governance systems across spatial scales and is more sensitive than other human activities^31^. Thus, we identified areas highly subject to human disturbance using the geographically gridded maps of the land system, which includes land use patterns and management practices (Supplementary Table 2). We summarized the mean risks of antibiotics for each watershed at four levels, as well as the mean land use composition and mean management intensity. Land use composition was reclassified into the area proportions of arable, built-up, and natural (mainly consisting of grassland and forestland) lands. Land management intensity was indicated by the manure application rate (characterized by manure nitrogen application rate per year, kg N/km^2^/yr) and irrigated area proportion because manure application and irrigation are generally considered the main pathways of antibiotics into soil^30^.

To analyze the responses of antibiotic pollution risks (response variable) to human activities using each generated dataset, we listed three land use composition indicators and two land management indicators as explanatory variables using linear regression models, while population and GDP were also considered socioeconomic indicators. We standardized the land use composition (area proportion of arable land) and management indicators via the minimum–maximum method. Land system intensification was estimated by summing the standardized scores of land use composition and management within a specific watershed.

### Scale effects and human impacts on the risk of antibiotics

The primary goal of this analysis was to identify the effects of human activities on antibiotic pollution risks when the spatial scale changes. The scale changes were characterized by (i) grain of scale: generating datasets by changing the grain size (that is, the watershed level at which data are generated), and (ii) extent of scale: resampling the datasets by changing the scale extent (that is, the proportion of sampled data to the whole dataset). To explore the scale effects, four watershed levels (sizes 3, 6, 9, and 12) were selected from the HydroSHEDS dataset^52^ to represent the changes in spatial grain (Supplementary Fig. 5); here, these levels were renamed levels 1, 2, 3, and 4, respectively. Watershed level 1 represented a broad-scale grain, while level 4 was a small watershed scale.

On the other hand, the extent of scale changes can generate a series of subdatasets. After we ranked the datasets according to increasing human footprints^53^, the subdatasets represented a gradient of human footprints. When the whole dataset across China was used, the large spatial extent only reflected the average condition and might consequently hide some local-specific relationships between land system intensification and antibiotic pollution risks to a small extent^54^. Thus, the model results based on the whole dataset could not completely represent the relationships between land system intensification and antibiotic pollution risks. To avoid arbitrary model choices, we conducted 20 runs by selecting different data to explore the effects of spatial extent change. Following the rank of whole datasets according to increasing human footprints, the scale extent was reduced through the omission of segments from the ranked datasets. We omitted 0–90% of the upper segments (in intervals of 10%) from the ranked datasets in sequence; that is, 0–90% of the watersheds with high human footprints were removed from the datasets in sequence and 10 subdatasets were generated (upper part omission, Supplementary Fig. 6c). The data included in the subdatasets represented the relationships between land system intensification and antibiotic pollution risks in watersheds with low human footprints. Then, we omitted 0–90% of the lower segments from the ranked datasets in sequence; that is, the watersheds with low human footprints were removed, and another 10 subdatasets were generated (lower part omission). The data included in these subdatasets represented the conditions in watersheds with high human footprints. Through this approach, 20 subdatasets with different ranges of human impacts and scale extents were generated. Then, the best models were selected via a procedure based on the corrected Akaike information criterion (AICc). We then considered the best models with ΔAICc <2, and this threshold can be up to 5 to include more than one plausible model^55^. Model averaging was conducted to calculate the coefficients of predictors and test their significance. This procedure was performed using the *dredge* function in the R package *MuMIn*^56^. Moreover, we characterized the contributions of land use and management to antibiotic-related risks. For doing so, we expressed the contributions of predictors as the percentage of variance explained, according to the absolute values of the ratio of the standardized regression coefficients to the sum of all standardized regression coefficients from all predictors.

### Risk–yield tradeoffs and thresholds for land system intensification

The crop yield data included rice, maize, wheat, and vegetable yields^57^. Then, generalized additive models (GAMs), which can be identified without prior subjective model specifications, were used to analyze the nonlinear relationships between antibiotic pollution risk (predictor variable) and crop yield (response variable). In the GAMs, nonparametric smoothers were used, and the basis dimension of the smoothing function was five^58^.

We calculated the risk–yield tradeoffs using the approach proposed for land management^59^. To remove the effects of differences in the measurement scale, we first standardized the two objectives: antibiotic pollution risk and crop yield, and the improvement (*P*) of land system intensification for one objective was estimated (antibiotic pollution risk is defined as one disservice):

1 $$P=\left({A}_{i}-{A}_{\min }\right)/\left({A}_{\max }-{A}_{\min }\right)$$

where *A*_*i*_ is the observed value of this objective in watershed *i*, and *A*_max_ and *A*_min_ are the maximum and minimum values in all watersheds, respectively. When the magnitude of improvement ranged from 0 to 1, a higher *P* value in watershed *i* represented a higher level for this objective in comparison with other watersheds, indicating that this objective could obtain more benefits from land system intensification in watershed *i*. Then, we quantified their tradeoffs in two dimensions. On the 1:1 line, improvement in crop yield equals improvement in antibiotic pollution risk, and their tradeoffs increase with distance from the 1:1 line. The risk–yield tradeoff is defined as the *P* value of crop yield subtracting the *P* value of antibiotic pollution risk, that is, the distance from the 1:1 line (Supplementary Fig. 9). When the tradeoff values were higher than zero, crop yield benefits were higher than antibiotic pollution risks.

The constraint line is the percentile boundary of the scattered point cloud between the response variable (antibiotic pollution risk, crop yield, and risk–yield tradeoff) and the constraint variable (i.e., land system intensification)^60^. Then, the value range of each response variable on the *x*-axis (constraint variable) of the scattered point cloud is divided into 50 parts to obtain 50 point columns (10 parts for the level-1 watershed). For the crop yields and risk–yield tradeoffs, we calculated the 99% upper boundaries as their constraint lines (i.e., chose the 99% quantile in each point column as the boundary point) and the 1% lower boundary for RQs as the constraint line of antibiotic pollution risks. The 99% upper boundary and 1% lower boundary were constructed to include most points of point clouds, indicating the maximum likelihood considering most scenarios. Due to nonlinearities, thresholds between constraint variables and response variables are often detected.

Using a moving-window approach, we analyzed the effects of land systems on antibiotic-related risks^61^. We generated a range of window sizes and calculated the relative range of RQs (the ratio of the range of RQs under a given window size to the maximum range under all window sizes). When the relative range exceeded 60%, we fixed the window size. For each window, we calculated the mean values of RQ, crop yield, and land system intensification (that is, area proportions of arable land, manure application rate, and irrigated area proportion). Then, we fitted risk–yield tradeoffs (*y*) against the land system variables (*x*) using inverted U-shaped curves as follows in Eq. (2):

2 $$y=a{x}^{2}+{bx}+c$$

where *a*, *b* and *c* are fitting parameters. The thresholds for land system variables were defined as *x* values when slopes were zero. That is:

3 $${thresholds}=-\frac{b}{2a}\,$$

### Reporting summary

Further information on research design is available in the Nature Portfolio Reporting Summary linked to this article.

### Supplementary information

Supplementary Information

Reporting Summary

Description of Additional Supplementary Files

Supplementary Data 1

Peer Review File

## Data Availability

The data of antibiotic pollution risks in soils from field work are provided in Supplementary Data 1. Other raw data of antibiotic pollution risks can be downloaded from *figshare*^44^. Livestock density can be obtained from GLW 3 dataset (https://www.fao.org/livestock-systems/en/). Gross domestic product, population density, and land use data are accessed in Resource and Environmental Science and Data Center (https://www.resdc.cn). Chemical fertilization and manure management data are collected from PANGAEA^62,63^. Pesticides data are available at *figshare*^64^. Irrigation data are available at https://luh.umd.edu. Soil data are publicly available from the following sources: soil physicochemical property data are collected from SoilGrids (SoilGrids.org), soil thickness data are available at ORNL DAAC^65^, and the saturated hydraulic conductivity data are collected from SoilKsatDB^66^. Groundwater table depth data can be requested from Fan et al.^67^. Terrain are collected from GTOPO30 Digital Elevation Model (https://lta.cr.usgs.gov/GTOPO30). Normalized difference vegetation index data are available at National Ecosystem Science Data Center, National Science & Technology Infrastructure of China (http://www.nesdc.org.cn). Crop production data are collected from 10.7910/DVN/PRFF8V.

## Code availability

The R codes for random forest model were adapted from Yang et al. (2021)^68^. Other codes for statistical analyses and visualization can be available from the corresponding author upon reasonable request.
