# Supplementary material for: Reducing risks of antibiotics to crop production requires land system intensification within thresholds
Source: Nat Commun. 2023 Sep 29;14:6094. doi: 10.1038/s41467-023-41258-x (PMC10541423; doi:10.1038/s41467-023-41258-x)
Supplement: Supplementary file 1 — Supplementary Information [file 41467_2023_41258_MOESM1_ESM.pdf]

Supporting Information for

Reducing risks of antibiotics to crop production requires land system intensification within thresholds

This file includes:

Supplementary Notes 1 to 2

Supplementary Figures 1 to 10

Supplementary Tables 1 to 2

Supplementary References

Supplementary Note 1 | Scale effect hypothesis

This section elucidates the approach for identifying models describing the relationships between human impacts and antibiotic pollution risk on different spatial scales. We assumed these relationships according to the development Kuznets curve hypothesis. Most cases featured an N-shaped or inverted U-shaped curve, showing a relationship between human societies and environments [1-3]. Using these two curves, we examined the effects of geographic scale on the influence of human activities on antibiotic pollution risk. We generated two maps according to the inverted U-shaped and N-shaped relationships (Supplementary Fig. 1). The maps provided information on the patterns of human impacts and antibiotic pollution risk in a given region. Supplementary Fig. 1a and Fig. 1c present the N-shaped and inverted U-shaped relationships, respectively.

The term scale has been defined variously in ecology and geography, but here, scale of a measurement describes the relative size or extent of space. In another word, scale is the extent (overall area) or grain (size of the individual units) at which a phenomena is studied (Supplementary Fig. 1e). To test the scale effects, we divided the maps into a series of cells with grain sizes 3 × 3 to 21 × 21. The generated relationship between human impacts and antibiotic pollution risk was generally consistent with the assumed relationship on the small scale (i.e., grain size = 3 × 3). However, with upscaling, the spatial grain influenced the generated relationships. On the large scale (i.e., grain size = 21 × 21), monotonous positive and negative relationships existed according to the assumptions of N-shaped and inverted U-shaped curves, respectively. Both assumptions generated opposite relationships, indicating that human activities did not necessarily jeopardise environmental sustainability from the spatial scale perspective. Compared with grain, spatial extent showed the limited effects on the relationships between human impacts and antibiotic pollution risk (mainly changed the relationships on the small extent, Supplementary Fig. 2). Thus, the reality of human impacts on antibiotic pollution risk across scales need to be addressed.

Supplementary Note 2 | Detection of antibiotics

Soil samples were collected from Ningbo city in Zhejiang and from central Yunnan. According to the land use, topographical features, and spatial locations, 238 experimental sites (1 m × 1 m) were established in Zhejiang (125 sites) and Yunnan (113 sites) (the georeferenced information is provided in Supplementary Data 1). At each experimental site, five to eight subsamples were randomly collected, and all subsamples were fully mixed. Grass, roots, stones, and other debris were removed from the soil samples. The composite soil samples were cold-stored and then sent to the laboratory. All of the samples were freeze-dried and stored at −20 °C until laboratory analysis.

Chemicals and standards. Target antibiotic standards, including tetracycline (TC), oxytetracycline (OTC), chlortetracycline (CTC), doxycycline (DOX), ofloxacin (OFL), norfloxacin (NOR), ciprofloxacin (CIP), enrofloxacin (ENR), and lomefloxacin (LOM), were obtained from Dr. Ehrenstorfer (Augsburg, Germany). Methanol, acetone, and acetonitrile were gradient-grade solvents used for liquid chromatography (Tedia Co. Inc., Fairfield, USA). All of the standards were dissolved in methanol and stored at −20 °C in the dark. Milli-Q water was produced using a purification system (Millipore, Billerica, MA, USA). A Na_2EDTA-McIlvaine buffer (pH was adjusted to 4) and methanol–acetonitrile–acetone solution (*v*:*v*:*v* = 2:2:1) were prepared [4, 5].

Pretreatment. First, 2 g of soil samples was added to 50 mL centrifuge tubes containing a mixture of 7.5 mL of Na_2EDTA-McIlvaine buffer and 7.5 mL of methanol–acetonitrile–acetone solution. Then, the tubes were vortexed (1 min) and ultrasonicated (20 min), followed by centrifugation (10 min, 3000 rpm). The procedure was conducted three times. The supernatants were combined and purified using glass fibre filters (0.22 μm, GF/F, Whatman, UK). The purified samples were preconditioned with 6 mL of acetone, 6 mL of methanol, and 6 mL of a 0.5 g/L Na_2EDTA solution (pH was adjusted to 3.5) and then concentrated using an Oasis HLB cartridge (6 mL, 200 mg, Waters, Milford, MA. USA). The cartridges were eluted with 3 mL of methanol and 5 mL of methanol (containing 1% formic acid). The leachates were evaporated to near-dryness under a gentle nitrogen stream, reconstituted to 1 mL with a high-performance liquid chromatography (HPLC) mobile phase, and stored at −20 °C until analysis.

Instrumental analysis. The antibiotics in samples were quantified via HPLC tandem mass spectrometry (Thermo Dionex Ulitmate 3000, USA) at a flow rate of 0.4 mL/min using a test column

(Waters Acquity UPLC BEH C_{18}, USA). The mobile phase consisted of eluent A (pure water with 0.2% methanol) and eluent B (methanol: acetonitrile, v/v = 4:6). The solution transitions for the gradient system were as follows: at time 0–0.5 min, 12% B; 0.5–6 min, 30% B; 6–9 min, 50% B; 9–9.5 min, 95% B; 9.5–12.5 min, 50% B; and 12.5–15 min, 12% B. For MS detection, the instrument was operated in the positive electrospray ionisation mode (ESI+) with multiple reaction monitoring.

Quality control and quality assurance. A spiked blank, a procedural blank, and a duplicate of soil were processed in parallel in each batch of 10 samples. The limit of detection (LOD) and limit of quantification (LOQ) for antibiotics were calculated according to the signal-to-noise ratios of 3:1 and 10:1, respectively. Spiking experiments were conducted to evaluate recovery in soils. One gram of soil sample was spiked with mixed standard solutions of antibiotics (100 ng dissolved in methanol). After the samples were mixed and aged for 24 h, they were extracted and analyzed via the same procedure as described above. Relative recovery was calculated using the following equation: recovery rate = $(C_s - C_b)/C_t \times 100\%$, where C_s is the concentration measured in the extract from a spiked soil sample, C_b is the concentration measured in the extract of a non-spiked fraction of the same soil sample, and C_t is the concentration of a standard solution with the same spiked amount. The average and relative standard deviations of relative recoveries were calculated from six replicate spiking experiments (Supplementary Fig. 10).

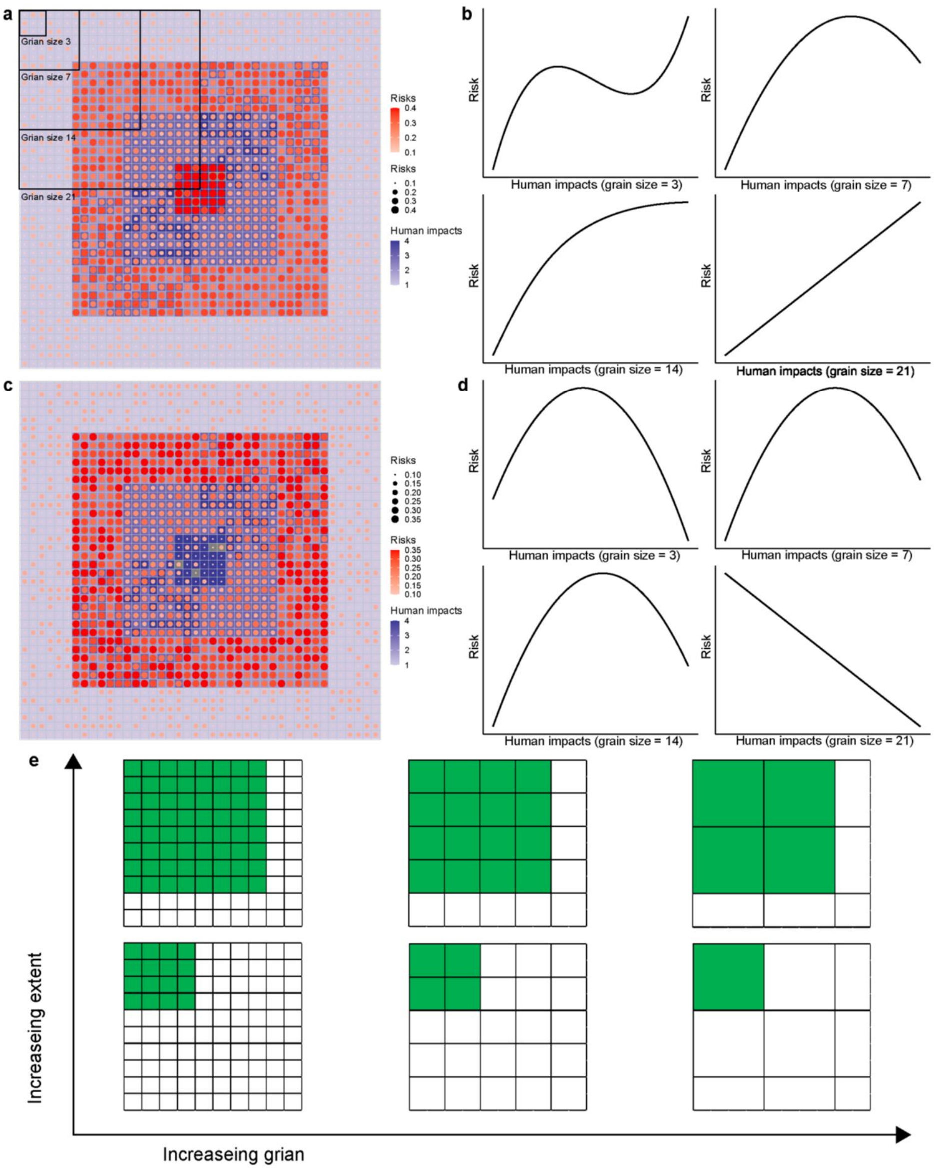

Supplementary Fig. 1 | Schematic of the main models of human impacts on antibiotic pollution risk on different scales. a, c, Assumed patterns of human impact intensity and antibiotic pollution risk, obtained using N-shaped (**a**) and inverted U-shaped curves (**c**). A larger size and number of red points represent a higher antibiotic pollution risk level, and the blue background represents human impact intensity. **b, d,** Potential models showing the relationships between human impacts and antibiotic pollution risk with increasing grain size, obtained using N-shaped (**b**) and inverted U-shaped curves (**d**). **e,** grain and extent comparison. Green pixels constitute the maps, with extent varying along the horizontal and grain varying along the vertical.

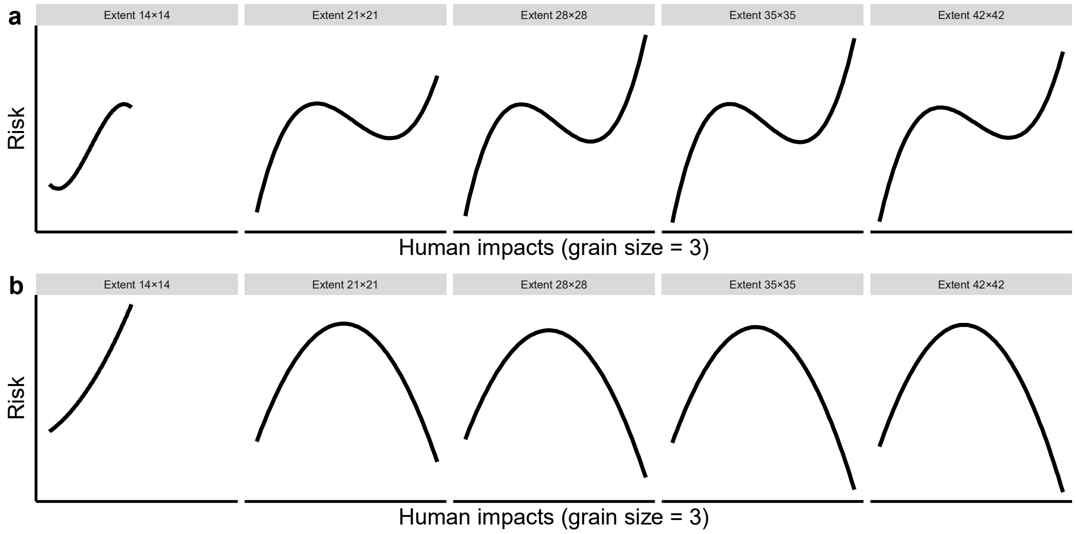

Supplementary Fig. 2 | The relationships between human impacts and antibiotic pollution risk with increasing extent. N-shaped curve (**a**) and inverted U-shaped curve (**b**).

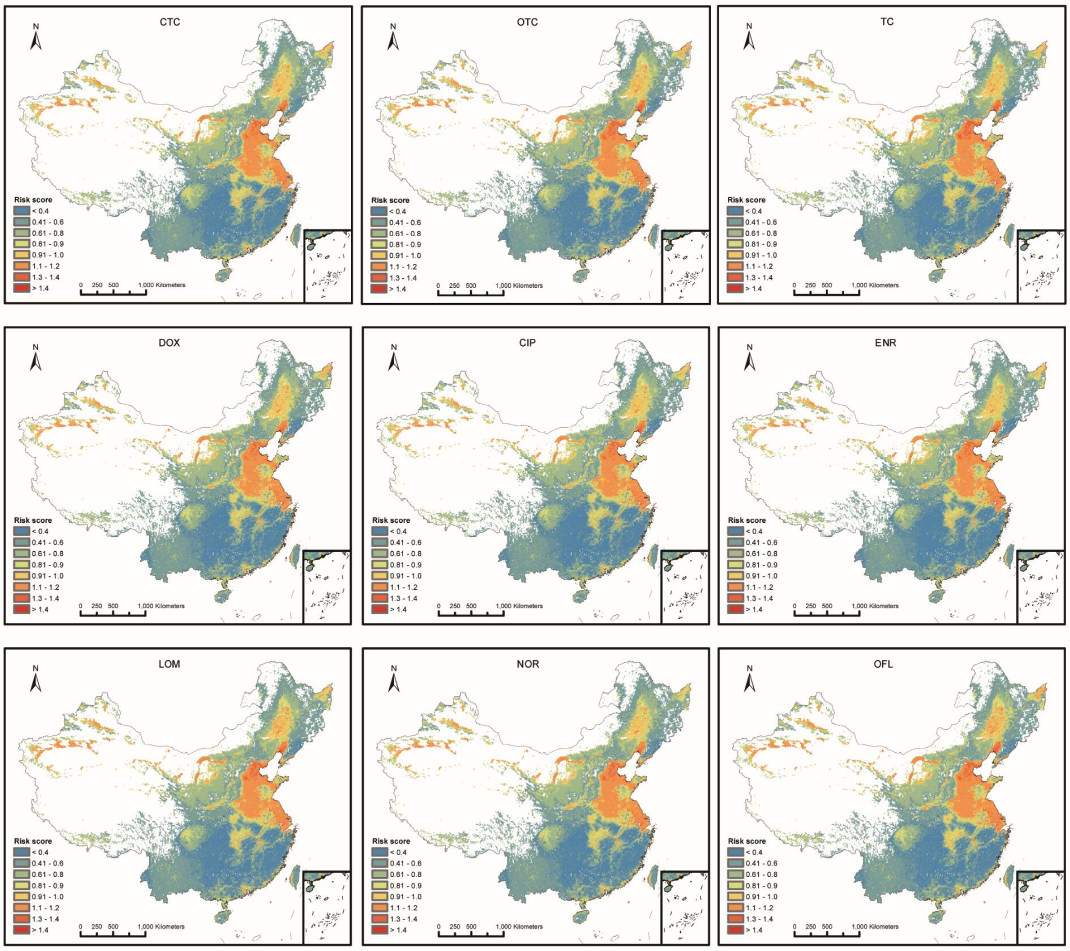

Supplementary Fig. 3 | Predicted risks of target antibiotics in Chinese soil. CTC: chlortetracycline, OTC: oxytetracycline, TC: tetracycline, DOX: doxycycline, CIP: ciprofloxacin, ENR: enrofloxacin, LOM: lomefloxacin, NOR: norfloxacin, OFL: ofloxacin.

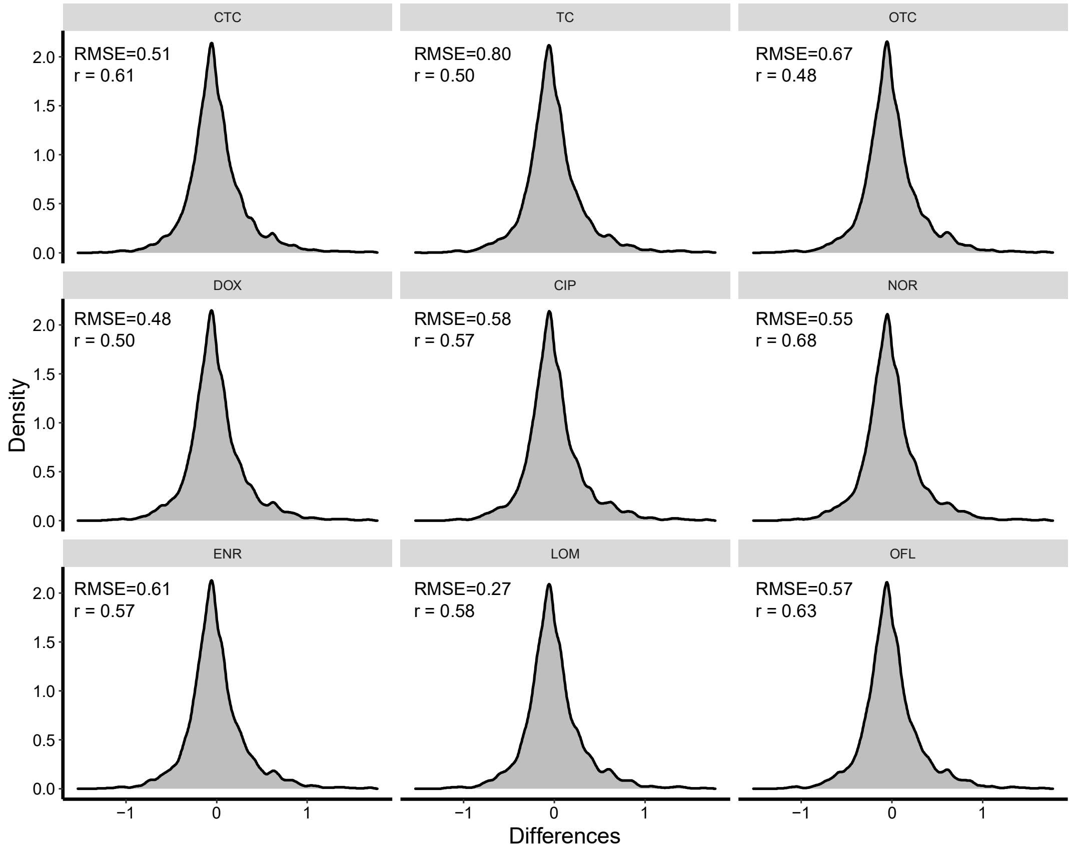

Supplementary Fig. 4 | Probability density of the differences between measured and predicted risks of target antibiotics in soil. CTC: chlortetracycline, OTC: oxytetracycline, TC: tetracycline, DOX: doxycycline, CIP: ciprofloxacin, ENR: enrofloxacin, LOM: lomefloxacin, NOR: norfloxacin, OFL: ofloxacin. Pearson's correlation coefficient (r) and root mean square error (RMSE) are used as metrics to validate the model prediction using validation datasets.

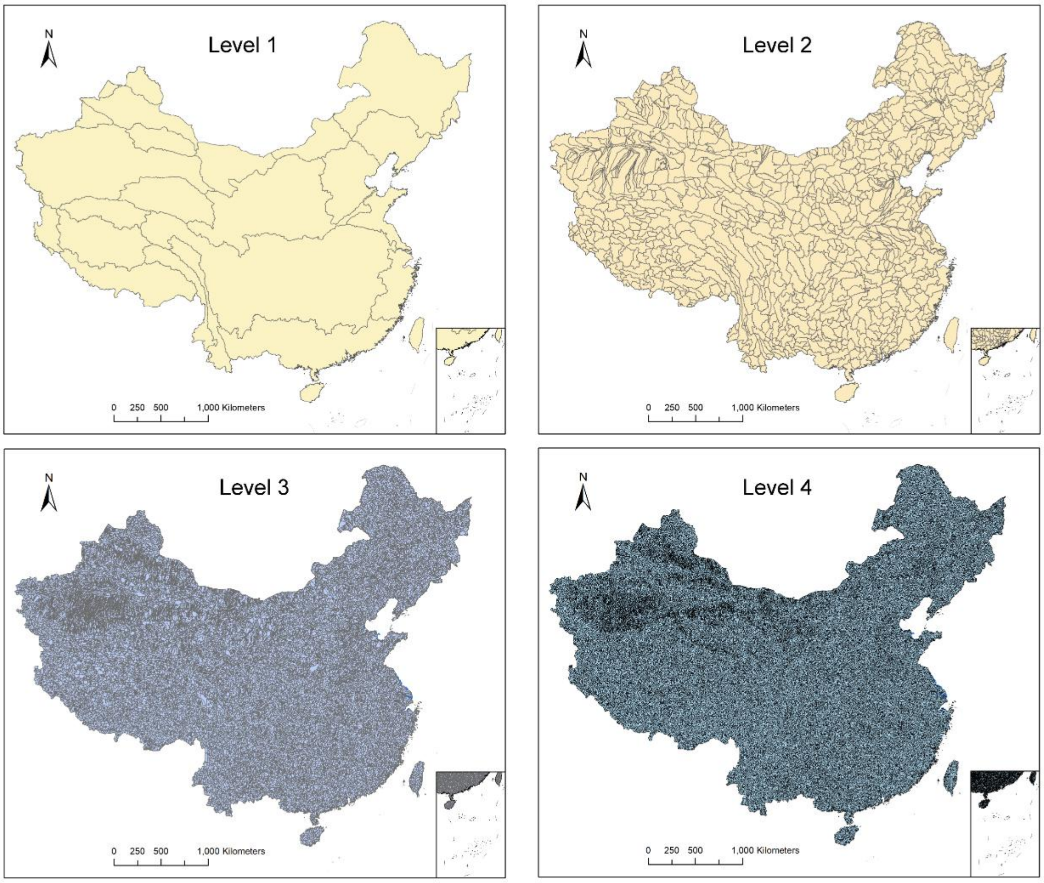

Supplementary Fig. 5 | Maps of watersheds in China on four spatial scales. Four levels of watershed (levels 3, 6, 9, and 12) were selected from the HydroSHEDS dataset [6] and were renamed levels 1, 2, 3, and 4, respectively. Watershed level 1 represents a broad-scale grain, while level 4 is a small watershed scale.

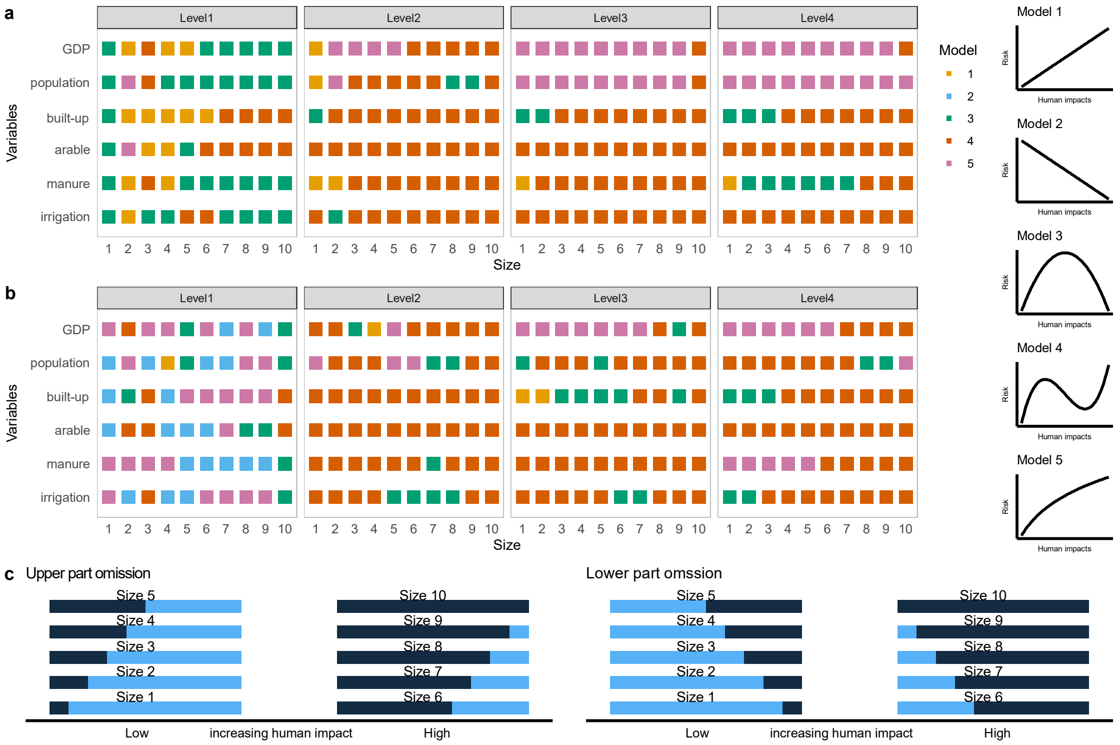

Supplementary Fig. 6 | Scale effects on human impacts on antibiotic pollution risk. a, b, Coloured squares indicate the model for 40 combinations (i.e., watershed size) and spatial extent (sampled segments); segments from the upper **(a)** and lower **(b)** limits of the human-impact gradient have been excluded. **c,** Schematic of changes in the spatial extent of scale. We omitted 0%–90% of upper and lower segments (the dataset was sorted according to increasing human footprints), as illustrated by the blue-shaded area of each horizontal column. The black-shaded area represents the sampled segments for analyses.

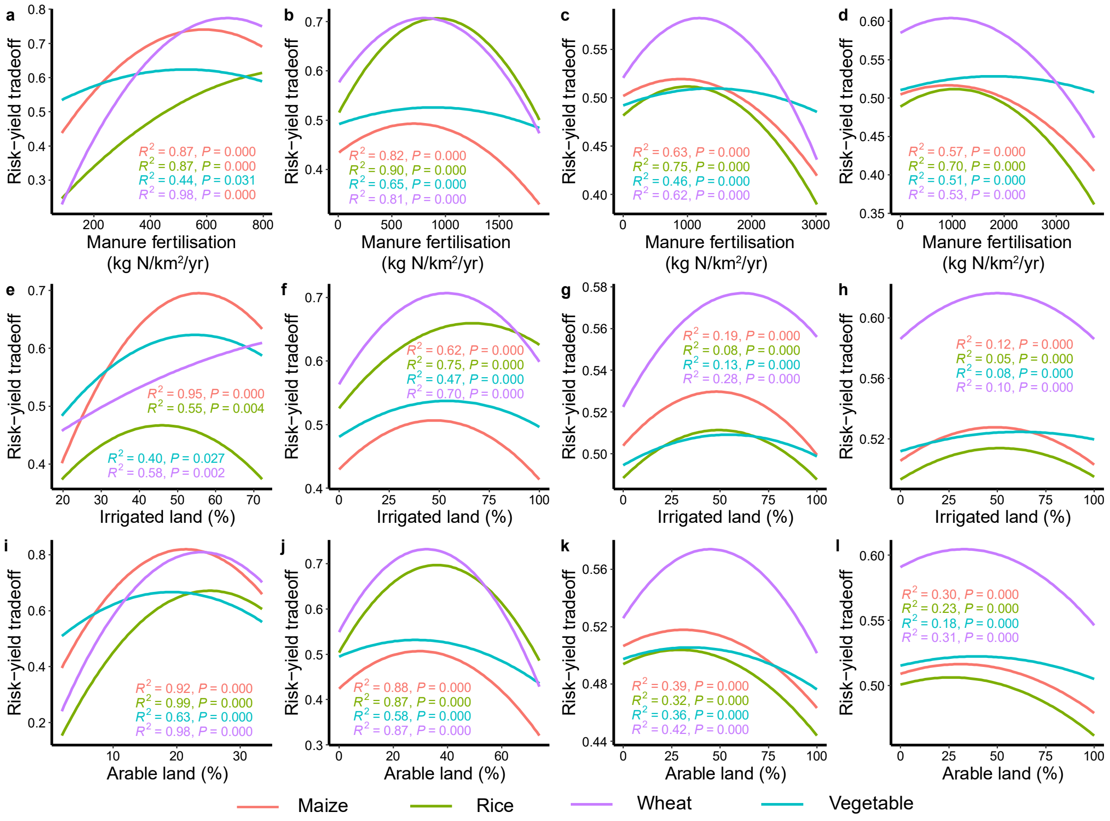

Supplementary Fig. 7 | Convex nonlinearities in relationships of risk-yield tradeoffs against land system intensification. The curves are predicted by generalized additive models. The data are generated using moving-window approach. Manure fertilization was characterized by manure nitrogen application rate (kg N/km^2 per year). The two-sided *p* values estimated by t-statistic are used to identify the statistical significance when their values are less than 0.05.

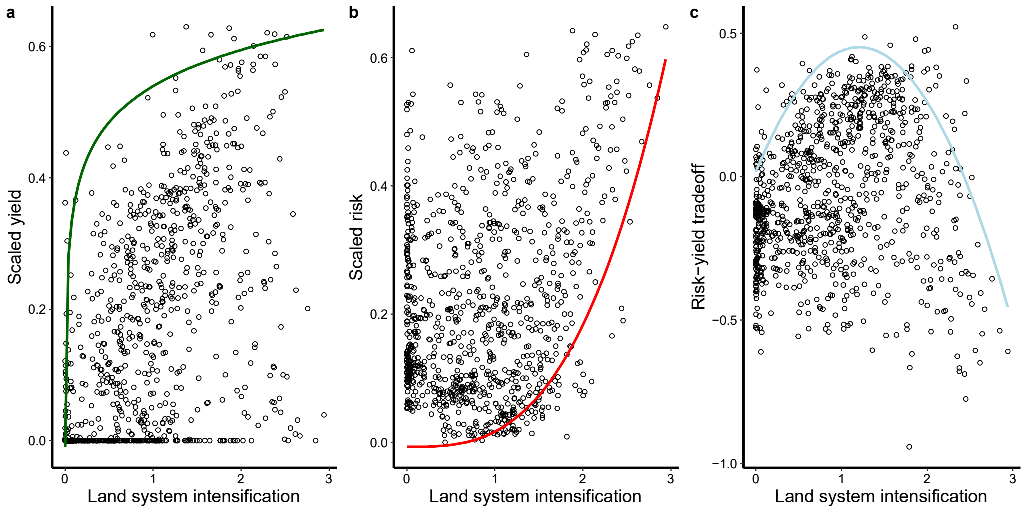

Supplementary Fig. 8 | 99% constraint lines for scattered point clouds. a, 99% upper constraint line for scaled rice yield at watershed level 2. **b**, 1% lower constraint line for scaled antibiotic pollution risks. **c**, 99% upper constraint line for scaled risk-yield tradeoffs. The 99% upper boundary and 1% lower boundary were constructed to include most points of point clouds, indicating the maximum likelihood considering most scenarios.

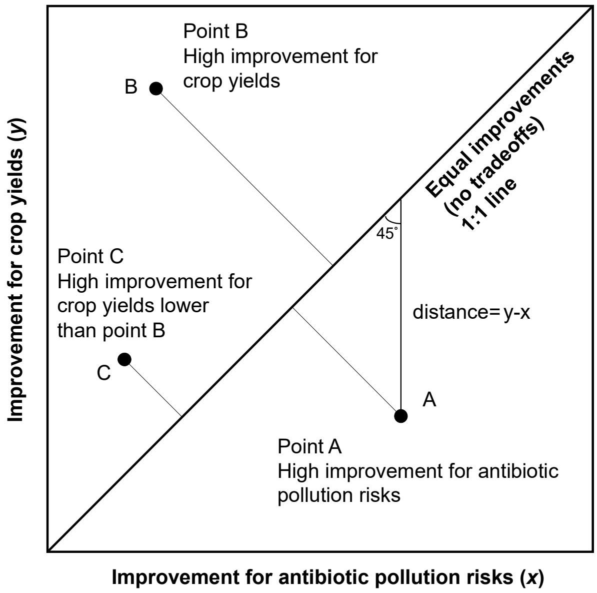

Supplementary Fig. 9 | Illustration and example of tradeoffs between antibiotic pollution risk and crop yield. A scaled value (antibiotic pollution risk or crop yield) indicates a higher improvement of land system intensification in a given watershed. We defined risk-yield tradeoff is defined as the scaled crop yield (y) subtracting scaled antibiotic pollution risk (x), that is distance from 1:1 line (that is, distance = $y - x$). When the scaled risk equals to scaled yield, they will on the 1:1 line. When scaled risk is higher than scaled yield, $y - x < 0$, i.e., point A. In contrast, when scaled risk is lower than scaled yield, $y - x > 0$, i.e., points B and C.

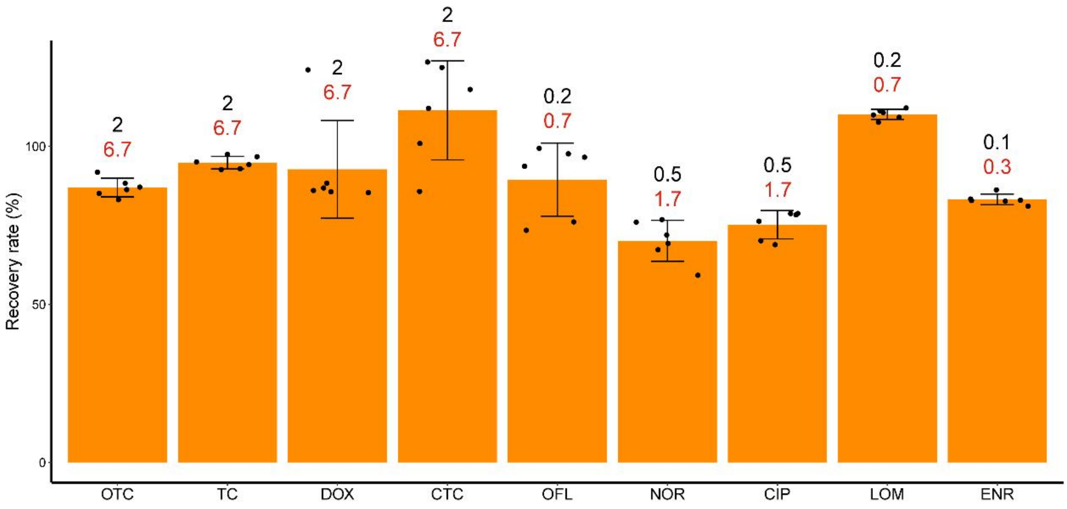

Supplementary Fig. 10 | The LODs, LOQs, and recovery of target antibiotics. The columns and error bars indicate mean recovery rates and standard deviations (n = 6 independent experiments). The numbers above the histograms are the LODs (black colour) and LOQs (red colour) of antibiotics (ng/g). The points show the data distribution. Chlortetracycline (CTC), oxytetracycline (OTC), tetracycline (TC), doxycycline (DOX), norfloxacin (NOR), ciprofloxacin (CIP), enrofloxacin (ENR), ofloxacin (OFL), lomefloxacin (LOM).

Supplementary Table 1 | Minimum ecotoxicity of target antibiotics to arable crops in soil environments. Other values are not presented, and can be found in published literatures [7, 8].

Compound	Arable crops	Test	Predicted no-effect concentration (μg/kg)	References
Oxytetracycline	Chinese cabbage	seed germination	20	9
Chlortetracycline	Chinese cabbage	seed germination	10	9
	Rice, Cucumber	-	300	10
	Rice	-	30000	10
	Cucumber seed	-	10000	10
	Cucumber root	-	7000	10
Tetracycline	Rice, Cucumber	seed germination	300	10
Doxycycline	Chinese cabbage	seed germination	10	9
Norfloxacin	Maize	root length	10.853	11
		shoot length	65.164	11
	Chinese cabbage	root length	13.888	12
		shoot length	1131.7	12
Ciprofloxacin	Maize	root length	7.97	13
		shoot length	11.23	13
	Chinese cabbage	root length	1.48	13
		shoot length	1.95	13
	Carrot; Lettuce	-	36	14
Enrofloxacin	Tomato	root length	89.07	15
		shoot length	98.53	15
	Cabbage	root length	4.61	15
		shoot length	6.08	15
	Cucumber	seed germination	910	10
		root elongation	910	10
	Wheat	seed germination	470	10
	Tomato	seed germination	950	10
Ofloxacin [a]	Algae	-	446.4	16
		-	3.503	17
Lomefloxacin [a]	Duckweed	wet weight	413.802	18
		frond number	494.856	18

a: The PNEC values are estimated from toxicity to aquatic plants, which are adjusted by equilibrium partition approach [19].

Supplementary Table 2 | Dataset of predictors used in this study.

Variables	Sources	Description
Livestock density	GLW 3 [20]	Expressed in total number in each pixel (5 arcmin), including cattle, pig, and chicken which are the main consumers of livestock antibiotics worldwide.
Gross domestic product (GDP)	Resource and Environmental Science and Data Center (https://www.resdc.cn)	Expressed in yuan/km^2, pixel size is 1 km.
Population density	Resource and Environmental Science and Data Center (https://www.resdc.cn)	Expressed in counts/km^2, pixel size is 1 km.
Land use	Resource and Environmental Science and Data Center (https://www.resdc.cn)	Pixel size is 1 km, land use type includes cropland, grassland, forest, water, impervious surface.
Chemical fertilization	Agricultural fertilizer use[21]	Nitrogen and phosphorous use which were expressed g/m^2/yr at a resolution of 0.5°.
Pesticides	PEST-CHEMGRIDSv1 [22]	The number of active ingredients applied on land, gridded at 5 arcmin resolution.
Manure management	Manure production and application [23]	Data are in ascii-format at a spatial resolution of 5 minutes (latitude by longitude). The unit is kg N/km^2/yr.
Irrigation	Land-Use Harmonization 2 [24]	Unit is fraction of crop area (%, resolution: 0.5°).
Climate	CRU TS [25]	Annual average precipitation and temperature data at 30-minute (0.5°) resolution were obtained from the Climatic Research Unit.
Soil	SoilGrids [26]	The soil properties indlude clay content (%), organic carbon content (kg/m^3), bulk density (kg/m^3). Spatial resolution is 1 km.
Soil thickness	Global 1-km Gridded Thickness of Soil, Regolith, and Sedimentary Deposit Layers [27]	This data set provides high-resolution estimates of the thickness of the permeable layers above bedrock (soil, regolith, and sedimentary deposits) within a global 30-arcsecond (~1-km). In this study, soil thickness is mainly considered.
Saturated hydraulic conductivity	SoilKsatDB [28, 29]	Expressed in cm/d, pixel size is 0.01°.
Groundwater	Groundwater table depth [30]	Expressed in [m]. Globally gridded at 15 arc-min resolution (30 km at the equator)
Terrain	GTOPO30 [31]	Elevation (m, resolution: 30 s), slope (°) was calculated based on the elevation.
Vegetation	National Ecosystem Science Data Center, National Science & Technology Infrastructure of China. (http://www.nesdc.org.cn)	Annual NDVI, pixel size is 30 m.

Crop production	SPAM [32]	42 crops yield, Pixel size is 0.083º.

Supplementary References:

1. Mao, F., et al. Inequality of household water security follows a Development Kuznets Curve. *Nat. Commun.* **13**, 4525 (2022)
2. Wu, X., et al. Decoupling of SDGs followed by re-coupling as sustainable development progresses. *Nat. Sustain.* **5**, 452-459 (2022)
3. Sarkodie, S.A. & Strezov, V. A review on Environmental Kuznets Curve hypothesis using bibliometric and meta-analysis. *Sci. Total Environ.* **649**, 128-145 (2019)
4. Zhou, X., Qiao, M., Wang, F.H. & Zhu, Y.G. Use of commercial organic fertilizer increases the abundance of antibiotic resistance genes and antibiotics in soil. *Environ. Sci. Pollut. Res.* **24**, 701-710 (2017)
5. Zhao, F., et al. Distribution, dynamics and determinants of antibiotics in soils in a peri-urban area of Yangtze River Delta, Eastern China. *Chemosphere* **211**, 261-270 (2018)
6. Lehner, B., Verdin, K. & Jarvis, A. New Global Hydrography Derived From Spaceborne Elevation Data. *Eos, Transactions American Geophysical Union* **89**, 93-94 (2008)
7. Pan, M. & Chu, L.M. Fate of antibiotics in soil and their uptake by edible crops. *Sci. Total Environ.* **599-600**, 500-512 (2017)
8. Carballo, M., Rodríguez, A. & de la Torre, A. Phytotoxic Effects of Antibiotics on Terrestrial Crop Plants and Wild Plants: A Systematic Review. *Arch. Environ. Contam. Toxicol.* **82**, 48-61 (2022)
9. Xiao, M.Y., An, J., Ji, Z.H., Cui, S. & Li, P. Toxic effects of six typical antibiotics on seed germination and physiological characteristics of Chinese cabbage. *Chinese Journal of Ecology* **33**, 2775-2781 (2014) (in Chinese).
10. Zhou, X., et al. Antibiotics in animal manure and manure-based fertilizers: Occurrence and ecological risk assessment. *Chemosphere* **255**, 127006 (2020)
11. Wang, P., Wen, B. & Zhang, S.Z. Phytotoxicity and Oxidative Stress of Norfloxacin on Maize（Zea mays L.）Germination Stage. *Asian Journal of Ecotoxicology* **5**, 849-856 (2010) (in Chinese).
12. Wei, Z.Y., et al. Ecotoxicity of Three Antibiotics to Shoots and Root Elongation of Cucumber, Rape and Chinese Cabbage. *Journal of Agro-Environment Science* **33**, 237-242 (2014) (in Chinese).
13. Li, T., et al. Toxicity of ciprofloxacin to three crops. *Asian Journal of Ecotoxicology* **8**, 442-446 (2013) (in Chinese).
14. Liu, Z., Zou, H., Lan, Z. & Li, X. Prioritized antibiotics screening based on comprehensive risk assessments and related management strategy in various animal farms. *J. Environ. Manage.* **319**, 115702 (2022)
15. Fu, B.M., et al. Inhibitory effect of combined pollution of enrofloxacin and Cu on root and shoot elongation of cabbage and tomato. *Asian Journal of Ecotoxicology* **10**, 157-163 (2015) (in Chinese).
16. Isidori, M., Lavorgna, M., Nardelli, A., Pascarella, L. & Parrella, A. Toxic and genotoxic evaluation of six antibiotics on non-target organisms. *Sci. Total Environ.* **346**, 87-98 (2005)

17. Backhaus, T., Scholze, M. & Grimme, L.H. The single substance and mixture toxicity of quinolones to the bioluminescent bacterium Vibrio fischeri. *Aquat. Toxicol.* **49**, 49-61 (2000)
18. Brain, R.A., et al. Effects of 25 pharmaceutical compounds to Lemna gibba using a seven-day static-renewal test. *Environ. Toxicol. Chem.* **23**, 371-382 (2004)
19. Zhao, F., et al. Soil contamination with antibiotics in a typical peri-urban area in eastern China: Seasonal variation, risk assessment, and microbial responses. *J. Environ. Sci.* **79**, 200-212 (2019)
20. Gilbert, M., et al. Global distribution data for cattle, buffaloes, horses, sheep, goats, pigs, chickens and ducks in 2010. *Sci. Data* **5**, 180227 (2018)
21. Lu, C. & Tian, H. Global nitrogen and phosphorus fertilizer use for agriculture production in the past half century: shifted hot spots and nutrient imbalance. *Earth Syst. Sci. Data* **9**, 181-192 (2017)
22. Tang, F.H.M., Lenzen, M., McBratney, A. & Maggi, F. Risk of pesticide pollution at the global scale. *Nat. Geosci.* **14**, 206-210 (2021)
23. Zhang, B., et al. Global manure nitrogen production and application in cropland during 1860–2014: a 5 arcmin gridded global dataset for Earth system modeling. *Earth Syst. Sci. Data* **9**, 667-678 (2017)
24. Hurtt, G.C., et al. Harmonization of global land use change and management for the period 850–2100 (LUH2) for CMIP6. *Geoscientifc Model Development* **13**, 5425-5464 (2020)
25. Harris, I., Osborn, T.J., Jones, P. & Lister, D. Version 4 of the CRU TS monthly high-resolution gridded multivariate climate dataset. *Sci. Data* **7**, 109 (2020)
26. Hengl, T., et al. SoilGrids250m: Global gridded soil information based on machine learning. *Plos One* **12**, e0169748 (2017)
27. Pelletier, J.D., et al. A gridded global data set of soil, intact regolith, and sedimentary deposit thicknesses for regional and global land surface modeling. *J. Adv. Model Earth Syst.* **8**, 41-65 (2016)
28. Gupta, S., Hengl, T., Lehmann, P., Bonetti, S. & Or, D. SoilKsatDB: global database of soil saturated hydraulic conductivity measurements for geoscience applications. *Earth Syst. Sci. Data* **13**, 1593-1612 (2021)
29. Dai, Y., et al. A global high-resolution data set of soil hydraulic and thermal properties for land surface modeling. *J. Adv. Model Earth Syst.* **11**, 2996-3023 (2019)
30. Fan, Y., Li, H. & Miguez-Macho, G. Global patterns of groundwater table depth. *Science* **339**, 940 (2013)
31. LDAAC. Global 30 Arc-Second Elevation Data Set GTOPO30. (2004).
32. Yu, Q., et al. A cultivated planet in 2010 – Part 2: The global gridded agricultural-production maps. *Earth Syst. Sci. Data* **12**, 3545-3572 (2020)
