# Supplementary material for: Reducing risks of antibiotics to crop production requires land system intensification within thresholds
Source: Nat Commun. 2023 Sep 29;14:6094. doi: 10.1038/s41467-023-41258-x (PMC10541423; doi:10.1038/s41467-023-41258-x)
Supplement: Supplementary file 3 — Description of Additional Supplementary Files [file 41467_2023_41258_MOESM3_ESM.pdf]

Title: Supplementary Data 1:

Description: This data have 238 records of risk quotient for antibiotics in soils of Zhejiang province and Yunnan province of China which are estimated from the measured environmental concentrations of antibiotics and risk assessment model.
