# Supplementary material for: Reducing risks of antibiotics to crop production requires land system intensification within thresholds
Source: Nat Commun. 2023 Sep 29;14:6094. doi: 10.1038/s41467-023-41258-x (PMC10541423; doi:10.1038/s41467-023-41258-x)
Supplement: Supplementary file 5 — Peer Review File [file 41467_2023_41258_MOESM5_ESM.pdf]

nature portfolio

Peer Review File

Reducing risks of antibiotics to crop production requires land system intensification within thresholds

Open Access This file is licensed under a Creative Commons Attribution 4.0 International License, which permits use, sharing, adaptation, distribution and reproduction in any medium or format, as long as you give appropriate credit to the original author(s) and the source, provide a link to the Creative Commons license, and indicate if changes were made. In the cases where the authors are anonymous, such as is the case for the reports of anonymous peer reviewers, author attribution should be to 'Anonymous Referee' followed by a clear attribution to the source work. The images or other third party material in this file are included in the article's Creative Commons license, unless indicated otherwise in a credit line to the material. If material is not included in the article's Creative Commons license and your intended use is not permitted by statutory regulation or exceeds the permitted use, you will need to obtain permission directly from the copyright holder. To view a copy of this license, visit http://creativecommons.org/licenses/by/4.0/.

Editorial Note: This manuscript has been previously reviewed at another journal that is not operating a transparent peer review scheme. This document only contains reviewer comments and rebuttal letters for versions considered at *Nature Communications*.

Reviewers' Comments:

Reviewer #1:
Remarks to the Author:
This is my second time to review this work. Happy to say that the authors have satisfactorily dealt with my concenrs and comments.
At the same time I I also understand the critics raised by other reviewers , however, I believe that the authors have introduced sufficient comments to address their concerns. So I hope this article has the impacts it deserves in the field.

Reviewer #2:
Remarks to the Author:
The authors have responded sufficiently well to our concerns and queries, and now they need to correct the grammar and edit the manuscript. For example, the abstract reads: "Crop production will substantially decreased". Please check and edit the entire text and figure and table captions.
In conclusion, this is an interesting study of an interesting subject. It lacks mechanistic analysis, but by being phenomenologically sound, I incline myself for acceptance now, unless there is not much space in the Journal.

Reviewer #1 (Remarks to the Author):

This is my second time to review this work. Happy to say that the authors have satisfactorily dealt with my concerns and comments.

At the same time, I also understand the critics raised by other reviewers, however, I believe that the authors have introduced sufficient comments to address their concerns. So I hope this article has the impacts it deserves in the field.

Response: Thank you very much for recommending our manuscript. We are delighted that the reviewer is satisfied with our revisions.

Reviewer #2 (Remarks to the Author):

The authors have responded sufficiently well to our concerns and queries, and now they need to correct the grammar and edit the manuscript. For example, the abstract reads: “Crop production will substantially decreased”. Please check and edit the entire text and figure and table captions.

In conclusion, this is an interesting study of an interesting subject. It lacks mechanistic analysis, but by being phenomenologically sound, I incline myself for acceptance now, unless there is not much space in the Journal.

Response: Thank you very much for your time and effort in reviewing our manuscript. According to suggestion of the reviewer, we have checked the grammar across the manuscript.
